# Accelerating the Production of Druggable Targets: Eukaryotic Cell-Free Systems Come into Focus

**DOI:** 10.3390/mps2020030

**Published:** 2019-04-16

**Authors:** Lena Thoring, Anne Zemella, Doreen Wüstenhagen, Stefan Kubick

**Affiliations:** Fraunhofer Institute for Cell Therapy and Immunology (IZI), Branch Bioanalytics and Bioprocesses (IZI-BB), Am Mühlenberg 13, D-14476 Potsdam, Germany; lena.thoring@izi-bb.fraunhofer.de (L.T.); anne.zemella@izi-bb.fraunhofer.de (A.Z.); doreen.wuestenhagen@izi-bb.fraunhofer.de (D.W.)

**Keywords:** in vitro translation, protein production, drug development, cell-free protein synthesis, eukaryotic lysates, microsomes, growth factors, enzymes

## Abstract

In the biopharmaceutical pipeline, protein expression systems are of high importance not only for the production of biotherapeutics but also for the discovery of novel drugs. The vast majority of drug targets are proteins, which need to be characterized and validated prior to the screening of potential hit components and molecules. A broad range of protein expression systems is currently available, mostly based on cellular organisms of prokaryotic and eukaryotic origin. Prokaryotic cell-free systems are often the system of choice for drug target protein production due to the simple generation of expression hosts and low cost of preparation. Limitations in the production of complex mammalian proteins appear due to inefficient protein folding and posttranslational modifications. Alternative protein production systems, so-called eukaryotic cell-free protein synthesis systems based on eukaryotic cell-lysates, close the gap between a fast protein generation system and a high quality of complex mammalian proteins. In this study, we show the production of druggable target proteins in eukaryotic cell-free systems. Functional characterization studies demonstrate the bioactivity of the proteins and underline the potential for eukaryotic cell-free systems to significantly improve drug development pipelines.

## 1. Introduction

Over the last decades, the pharmaceutical market has changed its focus from the production of small molecule components to more complex biopharmaceuticals. Biotherapeutics gain a growing share of the global pharmaceutical market [1] and newly developed products are based on therapeutical proteins such as monoclonal antibodies [2]. The rising importance of protein molecules for the pharmaceutical industry is not only present in the topic of biopharmaceuticals, but also in the case of drug development. Drug discovery and development is a costly and time-consuming process, which requires typically 12–15 years from an original idea to the launch of a final product [3]. Therefore one of the challenges in the pharmaceutical industry is to obtain optimal and efficient drug discovery pipelines [4]. The first step of drug discovery comprises the identification of a drug target and the vast majority of approved drugs are proteins [5]. Major drug target classes belong to antineoplastics, G protein-coupled receptors (GPCR’s), ion channels, kinases and proteases [5]. Following the process of identification, a target is further validated and possible hits are identified in a so-called lead discovery phase. The target validation techniques range from in vitro tools to knock down and knock out of the gene of interest to animal models mimicking the desired disease [6]. Current approaches for the validation are often time-consuming and cost inefficient [7]. Apart from this, the identification of hit molecules interacting with target proteins is based on screening approaches where a variety of efficient strategies exist [3]. A recombinant protein is often a prerequisite for most drug development strategies and this target protein needs to be produced in reliable systems. Nowadays, a huge number of protein production systems are available and used for drug discovery to evaluate the structure of disease-associated proteins and respective protein-protein interactions. Most of these systems are based on genetically engineered cell lines originating from pro- and eukaryotes. One of the best platforms for the production of soluble proteins is, for example, the pCold-glutathione S-transferase (GST) system, which is based on the prokaryotic expression host *Escherichia coli* [8]. Limitations of prokaryotic systems occur when complex mammalian target proteins requiring posttranslational modifications, cofactors and chaperons for correct protein folding, assembly and activity need to be produced. To circumvent these issues, eukaryotic cell-based expression systems are available, including yeast systems (*Pichia pastoris*, *Saccharomyces cerevisiae*, *Kluyveromyces lactis*) and mammalian systems (HEK293, Chinese hamster ovary cells (CHO cells)). Interestingly, mammalian systems are only rarely reported in screening literature [9]. Generation of eukaryotic stable cell lines for protein production purposes can be quite laborious as cells grow slowly, production time increases and protein yields are low thereby leading to costly protein production processes. Alternative protein production platforms, so-called cell-free protein synthesis systems were developed based on cell lysates instead of a whole, living cell. This technology emerged as a powerful and flexible tool for fast and efficient production of proteins whereby the surrounding environment can be easily adapted for the required approach [10]. Cell-free systems originating from different cellular hosts are currently available. The most commonly used cell-free system is based on *E. coli* cell lysates [11,12], which typically achieve up to 1 mg/mL of de novo synthesized protein. This system is already used for screening approaches in terms of the development of protein in situ arrays (PISA) [13] as well as nucleic acid programmable protein arrays (NAPPA) [14,15]. *E. coli* cell-free systems are limited in the performance of posttranslational modifications. Therefore, such systems are not suitable for the synthesis of complex mammalian proteins. This led to the development of the first eukaryotic cell-free protein synthesis system originating from rabbit reticulocytes. The rabbit reticulocyte system showed low translation efficiencies and posttranslational modifications of proteins can only be conducted by supplementing exogenous microsomes [16,17]. Over the years, a broad range of eukaryotic cell-free systems was developed exhibiting improved translational efficiencies and the opportunity to produce complex mammalian proteins due to the presence of endogenous microsomes [18]. Apart from the wheat germ cell-free system, which is characterized by a highly efficient translational machinery but limited in posttranslational modifications, eukaryotic cell-free systems based on yeast [19,20], insect cells [21], CHO cells [22], tobacco cells [23] and human cell lines [24] harbor endogenous microsomes. These microsomes are derived from the endoplasmic reticulum, thereby enabling a co-translational translocation of proteins and ER-based posttranslational modifications such as glycosylation, disulfide bridging and lipidation.

**Figure 1:**
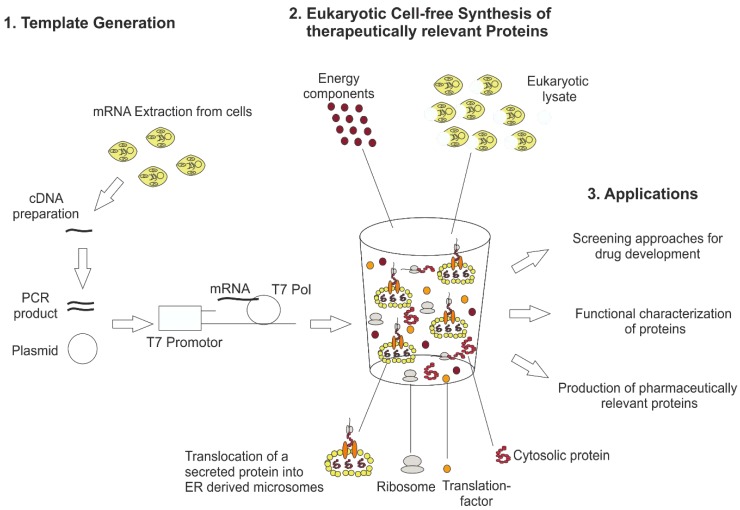
General principle of eukaryotic cell-free technology for research and therapeutical applications. For eukaryotic cell-free protein synthesis, a suitable DNA template is required, which can be directly prepared from cellular mRNA by RT-PCR. In this way, 5′ and 3′ regulatory sequences (T7 promotor, T7 terminator, stem loops and hairpin sequences) are added to the DNA template. Alternatively, plasmids harboring regulatory sequences can be used for eukaryotic cell-free protein synthesis. The DNA template is transcribed into mRNA using T7 RNA polymerase (T7 Pol) directly added to the cell-free synthesis reaction. Eukaryotic cell-free protein synthesis is based on a eukaryotic cell lysate including endogenous microsomes derived from endoplasmic reticulum. Special eukaryotic lysates like wheat germ and rabbit reticulocyte lysate do not include endogenous microsomes. The eukaryotic cell lysate is supplemented with previously produced mRNA and buffer and energy components to perform cell-free protein synthesis. Applications of eukaryotic cell-free protein synthesis are the development of novel screening platforms for drugs, the functional characterization of proteins and the production of biotherapeutics.

Despite significant advantages to produce challenging mammalian proteins, which the eukaryotic cell-free systems provide, they are typically not part of the drug discovery pipeline until now. In the past, eukaryotic cell-free systems were mostly cost-ineffective and characterized by low productivities, which made the technology inefficient for industrial applications. Immense development in the area of extract preparation, system optimization and reduction of process costs lead to well-established eukaryotic cell-free systems nowadays suitable for industrial applications. In this study, we demonstrate the production of druggable targets in eukaryotic cell-free systems. The general principle of these systems and future applications are pointed out in Figure 1. We started to produce the target proteins based on linear DNA templates and plasmids, transcribed DNA into mRNA in an in vitro transcription step using T7 RNA polymerase and used different eukaryotic cell-free systems for the production of the required proteins. The produced drug target proteins were functionally characterized to show a proof of concept for the application of the platform technology to the drug development pipeline.

## 2. Materials and Methods

### 2.1. Methods for Protein Production

This section comprises all methods and materials required for eukaryotic cell-free protein synthesis and a detailed analysis of the translational activity in eukaryotic cell-free systems. Various eukaryotic cell-free systems were applied and most of the results are derived in transcription/translation linked cell-free systems where transcription of mRNA and translation of a target protein are separated processes. The generation and design of required DNA templates are described initially, followed by the protocol for the transcription of mRNA from DNA templates and finally the method to perform eukaryotic cell-free reactions for the purpose of drug target production is depicted.

#### 2.1.1. Design and Generation of DNA Templates

DNA templates form the basis for eukaryotic cell-free synthesis. Special sequence characteristics enable the use of DNA templates as expression sequences for cell-free protein synthesis. 5′ and 3′ UTRs need to be adapted for eukaryotic cell-free synthesis including T7 promotor and T7 terminator sequences for the transcription reaction and stem-loops for an increased stability of mRNA. As described in Figure 1, depending on the application, linear as well as circular DNA templates can be applied directly to cell-free reactions. Special regulatory sequences are required for wheat-germ-based cell-free synthesis. The next section deals with the design and generation of optimal DNA templates.

#### Plasmids

Plasmids required for linear DNA template generation and cell-free protein synthesis were obtained from GeneArt (ThermoFisher Scientific, Regensburg, Germany). This included the following plasmids:

- Human telomerase (hTERT): pMA-hTERT-His, (*Sf*21 and CHO cell-free synthesis), pMA-hTR (*Sf*21, CHO and wheat germ cell-free synthesis)
- WNT: pcDNA3.1(+)-WNT3a, pcDNA3.1(+)-WNT5a, pcDNA3.1(+)-WNT5b, pcDNA3.1(+)-WNT6 (linear DNA template generation and *Sf*21 cell-free synthesis)

Further plasmid templates were cloned at Fraunhofer IZI-BB. This comprises the following plasmids:

- pIX4.0-Luc (*Sf*21 and CHO cell-free synthesis)
- pIVEX1.3-hTERT

All plasmids harbor the previously described regulatory sequences.

#### Generation of Linear Expression DNA Templates

Linear WNT DNA templates were generated by Expression PCR (EasyXpress Linear Template Plus Kit Plus, Biotech-Rabbit GmbH) to exchange the native signal peptide by a melittin signal peptide (Mel) and thereby increasing translocation efficiencies as reported by Kubick et al. [25]. The first step of template generation included gene (forward) and plasmid specific (reverse) primers amplifying the desired linear DNA template based on pcDNA3.1(+)-WNT plasmids. For the attachment of 5′- and 3′- regulatory sequences, a second PCR step was performed using a melittin signal sequence primer (N-Mel) in combination with an antisense primer (C-0). Primers used for the amplification steps are listed in Table 1. The preparation of the PCR procedure was performed according to the manufacturer’s instructions (EasyXpress Linear Template Plus Kit Plus, Biotech-Rabbit GmbH).

Mel-WNT3a harboring a C-terminal eYFP-tag (Mel-WNT3a-eYFP) was generated for the detection of cotranslational translocation of WNT-proteins into microsomal structures by confocal laser scanning microscopy. A two-step overlap extension (oe) PCR was performed for the amplification of WNT-eYFP fusion DNA templates. The first PCR step included a separate amplification of WNT-oe and oe-eYFP fragments. Gene-specific forward primer sequences and reverse primer sequences are listed in Table 2. During the second PCR step, both fragments were fused to each other. Furthermore, the 5′-end regulatory sequences and the melittin signal sequence were added using the adapter primer N-Mel and C-terminal antisense primer (C-SII). All PCR products were analyzed by 1% agarose gel electrophoresis and ethidium bromide staining. Primers were purchased from IBA (IBA GmbH, Göttingen, Germany).

#### 2.1.2. Transcription Reaction

The required mRNA for cell-free protein synthesis was prepared using an in vitro transcription approach. This technique is based on the application of a T7 RNA polymerase, which recognizes the T7 promotor of generated DNA templates for initiating mRNA transcription. In vitro transcription was carried out as described by Orth et al. [26] and transcription reaction was incubated for 2 h at 37 °C. Transcripted mRNA was purified prior to the application in cell-free translation reactions. Therefore, an intermediate gel filtration step is performed using a DyeEx 2.0 spin column (Qiagen, Hilden, Germany). The concentration of mRNA was analyzed using a Nanodrop 2000c spectrophotometer (ThermoFisher Scientific). Typically 1.0–1.3 nM of mRNA of different target proteins was added to translation reactions.

#### 2.1.3. Preparation of Eukaryotic Lysates

Three different eukaryotic lysates were applied to perform the cell-free synthesis of target proteins. Eukaryotic lysates were based on CHO cells, *Sf*21 cells and wheat germ and can be separated into microsome including (CHO and *Sf*21) and microsome absent (wheat germ) lysates. CHO and *Sf*21 lysates for eukaryotic cell-free synthesis were prepared at Fraunhofer IZI-BB. First, the appropriate cell lines (CHO-K1 (ECACC 85051005) and *Sf*21 (DSM ACC 119)) were cultured in a batch mode fermentation (equipment Sartorius AG). Process parameters (pO_2_, pH, temperature) were monitored and regulated to obtain a highly controlled lysate production process. Cells were harvested in the exponential growth phase when reaching a cell density between 4–6 ×10^6^ cells/mL using centrifugation. The cell pellet was washed with HEPES based buffer (100 mM HEPES-KOH, 100 mM KoAc, 4 mM Dithiothreitol (DTT)) to remove media components. A mild cell disruption procedure was performed using a 20-gauge needle and raw lysate was separated from cell debris and nuclei by centrifugation (10,000× *g* for 15 min at 4 °C). The raw lysate was further desalted and concentrated by size exclusion chromatography using a Sephadex G-25 column (GE Healthcare, Munich, Germany). Subsequently, A260 values were determined by Nanodrop 2000 (ThermoFisher Scientific) measurement and fractions with A260 values were pooled. Endogenous mRNA was digested by treating the pooled lysate with micrococcal S7 nuclease (10 U/µL, Roche, Penzberg, Germany). Nuclease activity was inhibited by the supplementation of 6.7 mM EGTA (ethylene glycol-bis(β-aminoethyl ether)-N,N,N′,N′-tetraacetic acid) (f. c.). Finally, CHO- and *Sf*21-lysates were aliquoted, shock-frozen in liquid nitrogen and stored at −80 °C. Wheat Germ lysate is commercially available in the form of RTS100 Wheat Germ CECF Kit (Biotechrabbit, Berlin, Germany).

#### 2.1.4. Eukaryotic Cell-Free Protein Synthesis

Individual eukaryotic cell-free protein synthesis systems differ in their composition and reaction conditions. *Sf*21- and CHO-based cell-free reactions have similar characteristics and were performed in a batch-formatted reaction mode. A 25 µL standard translation reaction of a *Sf*21 and CHO based cell-free synthesis was composed of 6 µL purified mRNA, 40% lysate, canonical amino acids (200 µM each), ATP (1.75 mM), GTP (0.45 mM) and ^14^C-labeled leucine (200 dpm/pmol) for the detection of de novo synthesized proteins. For the functional analysis of WNT proteins (β-catenin accumulation assay), proteins were synthesized in the absence of ^14^C-leucine. Protein translation reactions based on *Sf*21 lysates were incubated for 90 min at 27 °C, 600 rpm using a thermomixer (Eppendorf, Hamburg, Germany). Translation reactions based on CHO cell lysates were performed at 30 °C and 120 min with gentle shaking at 600 rpm. If required translation mixture (TM) of both cell-free reactions were further fractionated for analysis of protein translocation. The fractionation was realized by centrifugation at 16,000× *g* for 10 min at 4 °C in order to separate the ER-derived microsomal fraction (MF) of the cell lysate from the supernatant (S). The microsomal fraction was resuspended in PBS buffer without calcium and magnesium ions for further analysis.

To decrease the phosphorylation of eukaryotic translation factor eIF2α and thereby improving the capacity of cap-dependent translation initiation, the influence of the small component C38 (GSK2606414, GlaxoSmithKline, Dresden, Germany), a specific PERK inhibitor, was analyzed. Inhibitor containing lysate was preincubated for 10 min at RT before the cell-free reaction was started. To analyze the effect on the protein synthesis rate, the pIX4.0-Luc plasmid was added to the reaction and the yield of active luciferase was determined.

Synthesis in wheat germ lysate was performed in a transcription/translation coupled dialysis system using the RTS100 Wheat Germ CECF Kit (Biotechrabbit) for 24 h at 24 °C according to the manufacturer´s instructions. Again, ^14^C-leucine (2.47 dpm/pmol) was added to the reaction mixture to determine the size, yield and integrity of the de novo synthesized protein.

To obtain functional active hTERT enzyme, the assembly with a typical RNA component of telomerase is required [27]. The assembly was realized in two different ways: RNA was generated in a previous transcription reaction by using T7 RNA–polymerase followed by RNA purification using DyeEx spin columns (Qiagen). The freshly synthesized hTR RNA was directly added to the cell-free reaction (CHO and *Sf*21 cell-free reactions). Alternatively, a plasmid harboring the nucleotide sequence encoding the RNA component of human telomerase under control of the T7-promoter was added directly to the translation reaction (coupled reaction; wheat germ; final concentration of hTR plasmid: 20 ng/µL).

#### 2.1.5. Detection of eIf2 Phosphorylation

Western Blots analysis and ELISA were performed to monitor the presence of phosphorylated eukaryotic initiation factor eIF2 in eukaryotic cell-free systems. Phosphorylation of subunit α of eIF2 usually inhibits cap-dependent translation initiation in eukaryotic cells. This might also influence the translational activity of eukaryotic cell lysates. To enable the detection of eIF2α-P with western blot, the translation mixture of a CHO batch synthesis was acetone precipitated and present proteins were separated by SDS-PAGE according to the standard protocol (Section 2.2.3). After finishing the SDS-PAGE standard separation protocol, the gel was placed onto the “IBlot Gel Transfer Device” (ThermoFisher Scientific) and blotting was performed according to the manufacturer’s protocol. After finishing the blotting procedure, PVDF membrane containing desired proteins was washed three times in TBS for 10 min. Next, the membrane was blocked using 2% BSA in TBS/T at RT for 2 h or at 4 °C overnight followed by three times washing with TBS/T for 10 min. Subsequently, the membrane was incubated with gentle agitation for 3 h with the desired rabbit anti-eIF2α-P primary antibody (Cell Signaling Technology, Frankfurt, Germany) prediluted in a 2% BSA-TBS/T solution (1:1000). The previously described washing procedure was repeated and Anti-rabbit IgG, HRP-linked antibody (1:2000) (Cell Signaling Technology) was applied to the membrane. Conjugated horseradish peroxidase (HRP) enabled the detection of target protein bands. HRP substrate Amersham ECL select western blot detection reagent (Promega, Mannheim, Germany) was added to the membrane and incubated for 5 min. To visualize the specific protein bands chemiluminescence signal was analyzed using a Typhoon Trio + variable mode imager (GE Healthcare). A detection of eIF2α-P by ELISA was performed using a PathScan^®^ Phospho-eIF2α (Ser51) Sandwich ELISA according to the manufacturer’s protocol.

### 2.2. Qualitative and Quantitative Analysis of Cell-Free Synthesized Proteins

Methods and materials used for the analysis of cell-free synthesized proteins are described. In this section quantification, molecular size detection and functional analysis of the presented, cell-free synthesized proteins are introduced in detail.

#### 2.2.1. Luciferase Assay

For the optimization of translational activity and stability of eukaryotic cell lysates, a firefly luciferase was used as a model protein. Functional luciferase was quantified using a standard luciferase assay (Promega). Therefore, 5 µL of the cell-free translation mixture was applied to a white Nunc 96-well microtiter plate and mixed with 50 µL luciferase assay reagent. The luciferase assay was performed using a TriStar LB 941 multimode reader (Berthold Technologies, Bad Wildbach, Germany). The amount of functional protein was calculated based on the obtained relative light units (RLU), according to the following equation (calibration curve):

Active luciferase = 7 × 10^−5^ × RLU (1)

The amount of active luciferase was calculated from a triplet of three independent experiments (n = 3). Mean values and standard deviations were estimated as described in the TCA precipitation and scintillation measurement section.

#### 2.2.2. Quantification of Cell-Free Synthesized Protein Yield

Based on the incorporation of ^14^C-leucine in cell-free synthesized proteins, the respective protein yield can be estimated by scintillation measurement. Therefore, 5 µL aliquots of each translation mixture were mixed with 3 mL of a 10% (v/v) TCA-2% (v/v) casein hydrolysate solution (Carl Roth, Karlsruh, Germany) in a glass tube and incubated at 80 °C for 15 min. Afterwards, samples were chilled on ice for 30 min and retained on the surface of glass fiber filter papers (MN GF-3, Machery-Nagel, Düren, Germany) using a vacuum filtration system (Hoefer, Kleinblittersdorf, Germany). Filters were washed twice with 5% TCA and dried with acetone. Dried filters were placed into a scintillation vial (Zinsser analytic, Eschborn, Germany), 3 mL of scintillation cocktail (Quicksafe A, Zinsser analytic) was added and vials were agitated on an orbital shaker for at least 1 h. The scintillation signal was determined using the LS6500 Multi-Purpose scintillation counter (PerkinElmer, Berlin, Germany). The protein concentration was identified based on the obtained scintillation counts and protein specific parameters including molecular mass and amount of leucine. Error bars calculated for the protein yield show the individual standard deviation.

#### 2.2.3. SDS-PAGE and Autoradiography

The molecular size of radiolabeled, cell-free synthesized protein was analyzed using SDS-PAGE followed by autoradiography. First, 5 µL of the respective fraction of a cell-free synthesis reaction including the radiolabeled target protein was subjected to ice-cold acetone. Precipitated protein was separated by centrifugation (16,000× *g*, 4 °C, 10 min) and protein pellet was dried for at least 30 min at 45 °C. The dried protein pellet was dissolved in LDS sample buffer (ThermoFisher Scientific), heated at 70 °C for 10 min and loaded on a precasted NuPAGE 10% Bis-Tris gel (ThermoFisher Scientific). The gel was run at 185 V for 35 min according to the manufacturer’s protocol. Subsequently, gels were dried at 70 °C (Unigeldryer 3545D, Uniequip, Planegg, Germany), placed on a phosphor screen and radioactively labeled proteins were visualized using a Typhoon Trio + variable mode imager (GE Healthcare).

#### 2.2.4. β-Catenin Accumulation Assay

The functional activity of cell-free synthesized WNT protein (Mel-WNT3a) is analyzed based on canonical WNT signaling. In the applied assay, accumulation of β-catenin was analyzed using a western blot detection approach (described in Figure 3D). For this, confluently grown HeLa cells were stimulated with microsomes dissolved in cell culture media (DMEM, Merck Millipore, Darmstadt, Germany) with 1% Chaps (Sigma Aldrich, Taufkirchen, Germany) including cell-free synthesized Mel-WNT3a (50 ng/µL and 100 ng/µL). Recombinant WNT3a (RnD systems) and LiCl (50 mM, Sigma Aldrich) served as positive controls. To analyse background β-catenin expression, untreated HeLa cells (UTC) and stimulation with dissolved microsomes without cell-free synthesized WNT protein (NTC, volume equivalent to 100 ng/µL WNT3a volume) were used for the assay. After treatment of cells (3 h, 37 °C) with samples and control reagents, HeLa cells were lysed using RIPA-Buffer (50 mM Tris-HCl, 150 mM NaCl, 1% IGEPAL, 0.5% Sodium deoxycholate, 0.1% SDS, complete protease inhibitor cocktail tablets (Roche)). Cell lysates were further homogenized and centrifuged at 4000× *g* for 10 min at 4 °C. Supernatants were applied to a western blot. To enable comparability of the samples, total protein yield was determined using the Pierce BCA Protein Assay Kit (ThermoFisher Scientific) according to the manufacturer´s protocol. 20 µg of the total protein was loaded on a SDS-PAGE for further Western blot analysis (description of the method is in Section 2.1.5). Detection of β-catenin was realized using a primary rabbit anti-β-catenin antibody (1:1000) (Cell Signaling) and a secondary anti-rabbit HRP (Horseradish peroxidase) conjugated antibody (1:2000) (Cell Signaling Technologies). Detection of a β-actin was performed using a rabbit anti-β-actin antibody (1:1000) (Cell Signaling Technologies). The housekeeping protein β-actin served as an internal control and for normalization of image analysis.

#### 2.2.5. Analysis of Protein Translocation Using Confocal Laser Scanning Microscopy

In selected eukaryotic cell-free systems (*Sf*21 and CHO based system), microsomal structures are presently derived from the endoplasmic reticulum. These microsomes enable a direct translocation of secreted and membrane-embedded proteins followed by ER-based posttranslational modifications. In this study, the translocation of secreted eYFP fusion proteins (Mel-WNT-eYFP) into ER-derived microsomal structures was analyzed by confocal laser scanning microscopy (CLSM) using an LSM Meta 510 microscope (Zeiss, Oberkochen, Germany). The preparation of samples included a dilution of TM (5 µL) with PBS (15 µL) and a transfer of 20 µL diluted sample to µ-slides (Ibidi, Planegg, Germany). Samples were excited at 488 nm using an argon laser. The emission was detected using a band pass filter in the range of 500–550 nm.

#### 2.2.6. Telomerase Activity Assay

The functionality of telomerase was evaluated using the TeloTAGGG Telomerase PCR ELISA PLUS Kit (Roche). The general principle of this assay is illustrated in Figure 4B. In detail, 1 µL aliquots of the translation mixture were used for the amplification reaction where active telomerase adds telomeric repeats (TTAGGG) to the 3′-end of a biotin-labeled primer. The resulting products, harboring the telomere-specific repeats, were amplified again. In the next step, a 2.5 µL aliquot of the product was denatured and hybridized to a digoxigenin-(DIG)-labeled probe that recognizes telomeric repeats. The product consisting of telomeric repeats, the DIG-probe and the biotin-labeled primer were immobilized to a streptavidin-coated microplate and detected with a peroxidase-conjugated antibody against digoxigenin (anti-DIG-POD). By adding tetramethylbenzidine (TMB) substrate a colored complex was formed displaying an absorbance which could be measured at 450 nm with a reference wavelength of 690 nm. The relationship between the absorption of an internal standard and the sample gives the relative telomerase activity. Activity assays were performed in two independent experiments and standard deviations were calculated.

## 3. Results

In this section, the performance of eukaryotic cell-free systems for the production of druggable targets is exemplarily shown. The chapter starts with general strategies to obtain an optimal eukaryotic cell-free system for the synthesis of mammalian proteins. This is followed by two examples of drug target proteins, which are produced and functionally characterized in eukaryotic cell-free protein synthesis systems. In this case, secreted WNT-proteins and the cytosolically produced hTERT enzyme is chosen to show the potential of the system for future protein characterizations and drug development.

### 3.1. Strategies to Optimize Protein Production in Eukaryotic Cell-Free Systems

**Figure 2:**
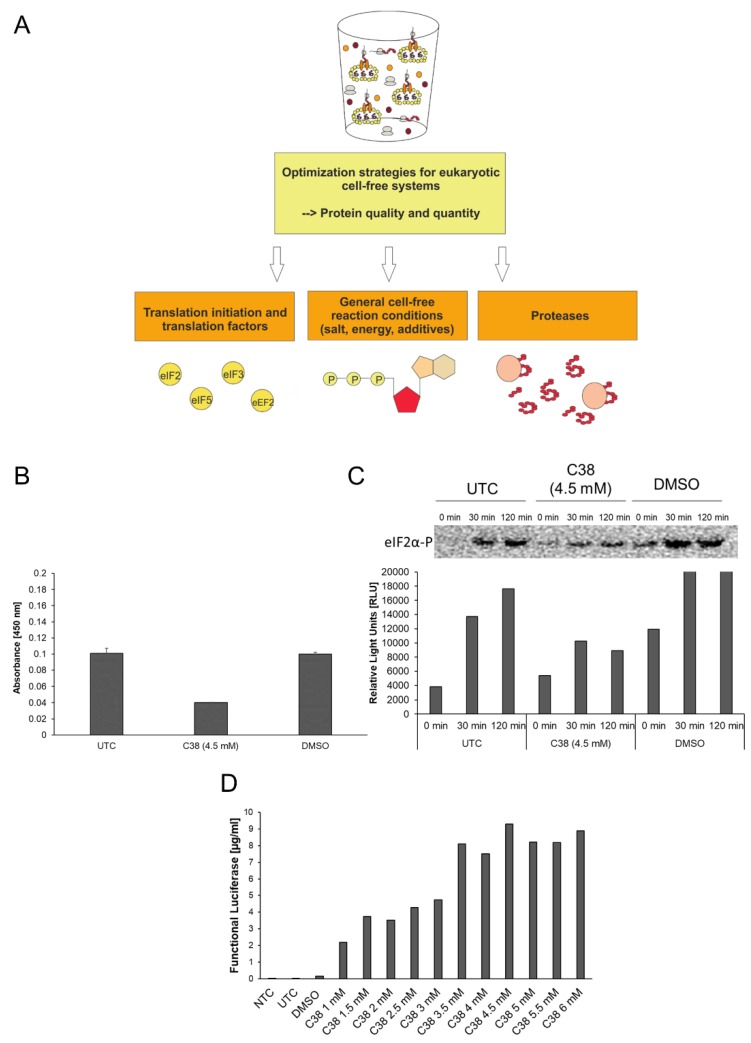
Optimization strategies for eukaryotic cell-free systems. (**A**) Schematic overview of strategies for improvement of eukaryotic cell-free systems concerning protein quality and quantity. (**B**) PathScan^®^ Phospho-eIF2α (Ser51) Sandwich ELISA (Cell Signaling) for the detection of eIF2α-P in CHO cell-free reactions. Untreated control (UTC), DMSO treated samples (DMSO) and C38 treated samples were analyzed for the presence of eIF2α-P. ELISA was performed according to the manufacturer´s protocol. (**C**) Western Blot analysis of UTC, DMSO treated samples (DMSO) and C38 treated samples using a primary anti-eIF2α-P antibody (Cell Signaling) and a secondary anti-rabbit-HRP antibody. Western blot signal was detected using ECL reagent (Promega) and a Typhoon Trio Plus Imager (GE Healthcare). Image analysis was performed using ImageQuant TL software (GE Healthcare). (**D**) Eukaryotic cell-free reaction in the presence of C38 PERK inhibitor. CHO lysate based cell-free reaction was performed using pIX4.0-Luc plasmid (Luciferase) in the presence of various concentrations (1 mM up to 6 mM) of small component C38. Production of cell-free synthesized Luciferase was analyzed using a standard Luciferase assay (Promega).

Protein translation, stability and preservation require certain conditions, which need to be realized in cell-free protein synthesis systems. In this study, eukaryotic cell-free protein synthesis systems are applied to produce drug target proteins. There are several weak points which can be addressed for the improvement of the eukaryotic cell-free systems. A general overview of the optimization strategies is pointed out in Figure 2A. In general, three main topics for the optimization of the systems are available. First, translation factors need to be present in a highly active form in the eukaryotic lysate. Cell cultivation and lysate preparation can influence the activity of translation factors due to nutrient limitations, oxidative stress and initiation of unfolded protein response. Partial inactivation of translation factors limits the production rate of eukaryotic cell-free protein synthesis systems. In addition, proteolytic enzymes in eukaryotic lysate can influence the stability of target proteins and translation associated factors. A general and targeted inhibition of proteases can improve cell-free systems. An example of the effective use of protease inhibitors is depicted by the application of caspase inhibitors in eukaryotic continuous exchange cell-free systems [22,28]. Apart from the lysate itself, general reaction conditions can be improved by adjusting energy components, buffer conditions and salt concentrations.

In Figure 2, the topic of translation initiation factors is addressed in the context of cap-dependent translation initiation in eukaryotic cell-free systems. Eukaryotic translation initiation is a complex process that requires the presence of numerous translation initiation factors in an active state. Inactivation of translation is a regulative tool in living cells to react to stress responses. One of the main initiation factors in terms of regulatory effects is the eukaryotic initiation factor 2 (eIF2). eIF2 can be subdivided into three subunits (α, β, γ), while phosphorylation of subunit α leads to factor inactivation and thereby inhibition of cap-dependent translation initiation. In Figure 2B–D activity of translation factor eIF2 was analyzed and factor phosphorylation was investigated in the presence of the specific phosphorylation inhibitor small component C38 (GSK2606414, GlaxoSmithKline). C38 specifically inhibits the protein kinase R (PKR)-like endoplasmic reticulum kinase PERK, one of the kinases responsible for eIF2α phosphorylation. For the experimental setup, cell-free reactions based on CHO cell lysate were used. eIF2α-P specific ELISA (Figure 2B) and western blot analysis (Figure 2C) were performed using untreated cell-free reactions (UTC), C38 treated cell-free reactions and DMSO treated cell-free reactions. DMSO, the solvent for C38, serves as a background control for eIF2α-P. ELISA results displayed an absorbance of 0.1 units in the UTC and the DMSO treated sample (Figure 2B). A decrease in absorbance was detected in the C38 treated sample (0.04 units). Similar results were obtained in western blot analysis (Figure 2C). Western blot analysis was performed using cell-free reaction samples taken at different time points of cell-free reaction (0 min, 30 min, 120 min). After 30 min and 120 min reduced intensities of eIF2α-P samples were detected after treatment with C38 in comparison to UTC and DMSO samples. The intensity of phosphorylation increases over time in UTC and DMSO samples. The result of both analyses depicts how the treatment of eukaryotic cell-free reaction with C38 led to a decrease in eIF2 phosphorylation. The following results show the influence of the inhibitor on the protein production rates (Figure 2D). Model protein Luciferase was chosen to evaluate the productivity of the system in the presence of C38. The yield of active Luciferase was evaluated using Luciferase detection reagent and chemiluminescence analysis. An NTC consisting of eukaryotic cell-free reaction without synthesized protein was prepared as a background control for the assay. A significant increase in protein yield was detected by treatment with C38. While UTC and DMSO treated samples show low protein yields in the range of 0.05 µg/mL–0.1 µg/mL an increase up to 9.5 µg/mL was detected using 4.5 mM of C38. A saturation of protein yield was reached using 4.5 mM C38. These results underline the beneficial effect of C38 on the translational activity of eukaryotic cell lysates.

### 3.2. Synthesis of WNT Proteins in Sf21 Cell-Free Systems

**Figure 3:**
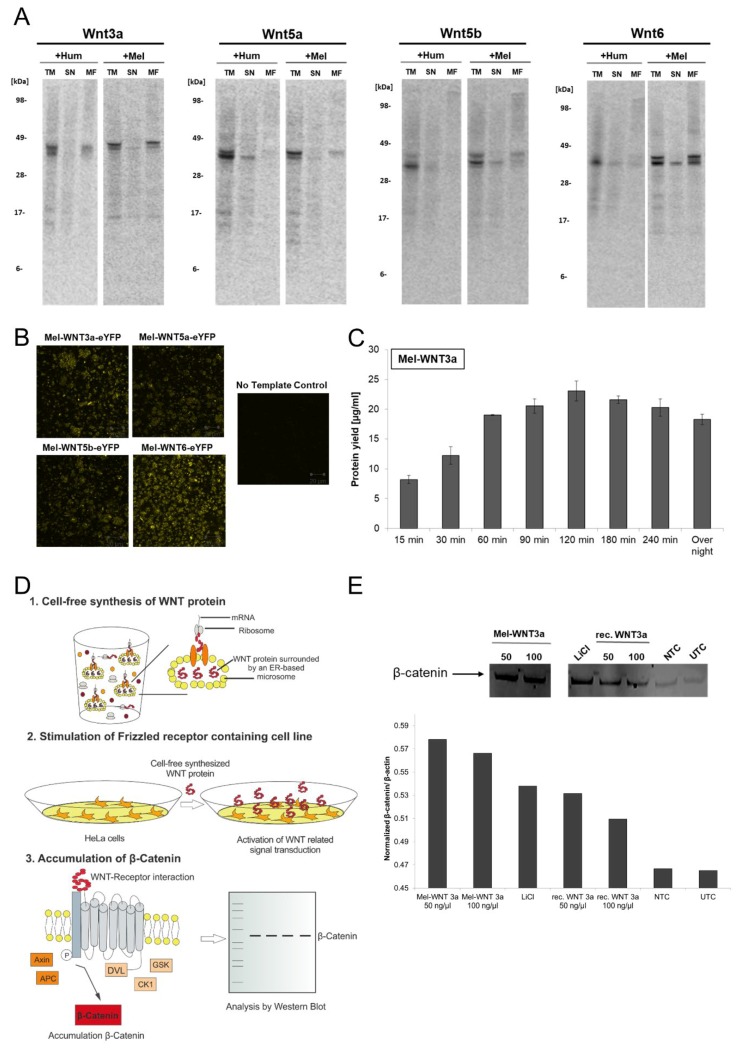
Cell-free synthesis of WNT proteins using *Sf*21 cell lysate. WNT proteins were synthesized in a batch-formatted *Sf*21 cell-free system. (**A**) Autoradiography of cell-free synthesized WNT proteins (WNT3a, WNT5a, WNT5b, WNT6). The qualitative analysis shows the expected molecular weight of the produced proteins. Production of WNT types was carried out with a human signal peptide (on the basis of pcDNA3.1(+)-WNT) and with a melittin signal peptide (on the basis of Expression PCR product). Translation mixture (TM) of cell-free synthesized WNT proteins were separated by centrifugation into a supernatant fraction (SN) and microsomal fraction (MF). (**B**) Analysis of translocation of WNT-eYFP fusion proteins by confocal laser scanning microscopy. (**C**) Analysis of Mel-WNT3a protein yield in a time course experiment. Radiolabeled proteins were TCA precipitated, transferred to a filter paper and dissolved in scintillation cocktail. Protein yield was determined by scintillation measurement using an LS6500 multi-purpose scintillation counter (PerkinElmer). (**D**) Schematic overview of the assay for functional characterization of WNT proteins based on canonical WNT signaling. (**E**) Functional characterization of cell-free synthesized Mel-WNT3a (50 ng/µL and 100 ng/µL) using the previously illustrated assay. Western blot analysis of accumulated β-catenin using primary anti-β-catenin and secondary anti-rabbit-HRP antibody (Cell Signaling). Protein band was detected using an ECL reagent (Promega) and a Typhoon Trio Plus Imager (GE Healthcare). Image analysis was performed with a housekeeping gene (β-actin) for normalization using ImageQuant TL software (GE Healthcare). NTC: No template control (cell-free reaction without WNT DNA template).

WNT proteins belong to a family of signaling proteins which are essential for many biological processes. This highly conserved family of signaling molecules [25] is involved in body axis formation, embryonic growth, cell differentiation and proliferation as well as tissue homeostasis [29,30]. WNT signal transduction is one of the most important pathways in human development and maintenance. Thus, dysfunction of WNT pathway regulation in human organisms plays an important role in the formation of well-known diseases [31] including various cancer types, especially colon and lung cancer, diabetes, kidney disorders and neurodegeneration [32,33,34]. WNT proteins are secreted proteins harboring several posttranslational modifications (glycosylation, lipidation). Such modifications are mandatory to maintain the activity of the proteins. Research in the field of WNT signal transduction is often limited by the inaccessibility of the produced bioactive WNT proteins [35,36]. Therefore novel production systems are required to produce WNT proteins to simplify future drug development. In this chapter, the production of WNT proteins in eukaryotic cell-free systems (*Sf*21 cell-free system) is described. In the first set of experiments, four candidates of WNT proteins (WNT3a, WNT5a, WNT5b, WNT6) were selected (Figure 3A). WNT proteins were cell-free synthesized in the presence of ^14^C leucine to enable further quantification and estimation of molecular weight by autoradiography. Two variants of the selected WNT proteins were synthesized and analyzed by autoradiography: The first variant harbors the original human WNT sequence including the human signal peptide, the second variant contains a melittin signal peptide instead of the human signal peptide. Previous studies showed an efficient translocation of secreted proteins into microsomes of *Sf*21 cell-free systems using the melittin signal peptide [37]. The exchange of human signal peptide to melittin signal peptide was realized by Expression PCR. Subsequently, PCR products were directly applied to the cell-free reaction. Prior to analysis, cell-free TM containing WNT proteins was separated into a SN fraction and MF to analyze the translocation of proteins into ER-derived microsomes. The apparent molecular mass of cell-free synthesized WNT ligands (listed in Table 3) was identified by SDS-PAGE followed by the autoradiography of radiolabeled proteins. Different types (WNT3a, WNT5a, WNT5b, WNT6) and variants (+Hum/+Mel) of WNT proteins showed bands at the expected molecular mass in the range of 39 kDa to 43 kDa (Figure 3A). In the TM and MF fractions, additional protein bands were detected displaying a slightly higher molecular mass than the expected protein bands, thereby indicating posttranslational modifications like glycosylation. Glycosylation of WNT ligands was further confirmed by PNGaseF treatment of TM samples (Supplementary Figure S1). The endoglycosidase treatment led to a disappearance of higher molecular mass bands on the autoradiograph thereby indicating the presence of glyco-modifications. Supernatant fractions of WNT proteins only harbor a single protein band at the expected molecular mass. Moreover, WNT protein variants containing a melittin signal peptide showed an increase in the intensity of protein bands in the microsomal fraction compared to WNT proteins with a human signal peptide. Therefore, autoradiography data imply differences in protein translocation into microsomes. In this case, melittin signal peptide also showed a more efficient translocation into microsomes of *Sf*21 cell-free systems. The translocation of WNT proteins into ER-derived microsomes was further verified by CLSM (Figure 3B). For this, suitable DNA templates for Mel-WNT-eYFP fusion proteins (WNT3a, WNT5a, WNT5b and WNT6) were generated to enable the visualization of protein translocation into ER-based microsomes. After finishing an *Sf*21 based cell-free synthesis of WNT-eYFP variants, pre-diluted translation reaction was analyzed by CLSM. To verify background fluorescence of the cell-free translation mixture, a NTC was prepared without any cell-free produced protein. A strong localization of the fluorescence signal in vesicle-like structures was detected for all WNT variants but lacked in the NTC sample, as expected. These results give a hind for successful translocation of WNT proteins into ER-based microsomes.

For further characterization of cell-free synthesized WNT proteins, Mel-WNT3a was exemplarily chosen to show the analysis of cell-free reaction and functional characterization. Figure 3C showed the obtained protein yields of Mel-WNT3a in *Sf*21 lysate based cell-free systems. After 120 min reaction time, the maximum protein yield of 23 µg/mL Mel-WNT3a was obtained. Longer reaction times led to a slight degradation of WNT protein. On the basis of this result, a functional characterization of Mel-WNT3a was performed. The general principle of the applied assay is based on the activation of the canonical WNT signaling pathway. During canonical WNT signaling, the WNT protein binds to the cell membrane receptors LRP5/6 and Frizzled and thereby activates intracellular signaling processes. This leads to the accumulation of β-catenin, a transcription regulator which actives WNT specific gene expression. The schematic protocol of the assay is illustrated in Figure 3D. The microsomal fraction harboring cell-free synthesized Mel-WNT3a is treated with 1% Chaps in cell culture medium to release translocated WNT proteins. The dissolved cell-free-WNT mixture (50 ng/µL and 100 ng/µL) is directly applied to HeLa cells and after 3 h of incubation, cells are lysed and accumulation of β-catenin is evaluated by western blot analysis. Apart from stimulation with cell-free produced WNT protein, control simulations were performed using cell-free reaction without synthesized protein (NTC), recombinant commercially available WNT protein (rec. WNT3a (50 ng/µL and 100 ng/µL)) and LiCl as a signaling stimulator (LiCl). UTC was taken as a background control. Results from the western blot analysis (Figure 3E) show the highest intensity of the β-catenin protein band by applying cell-free produced WNT-protein as a stimulant. Increased intensities were also detected for recombinant WNT and LiCl in comparison to the NTC and UTC sample. Image analysis where a β-actin housekeeping gene was used for normalization confirm these results.

The obtained results for the production of WNT proteins in *Sf*21 cell-free systems underline the potential for the fast production of complex and functional signaling proteins in eukaryotic cell-free systems.

### 3.3. Eukaryotic Cell-Free Systems for the Production of Human Telomerase

**Figure 4:**
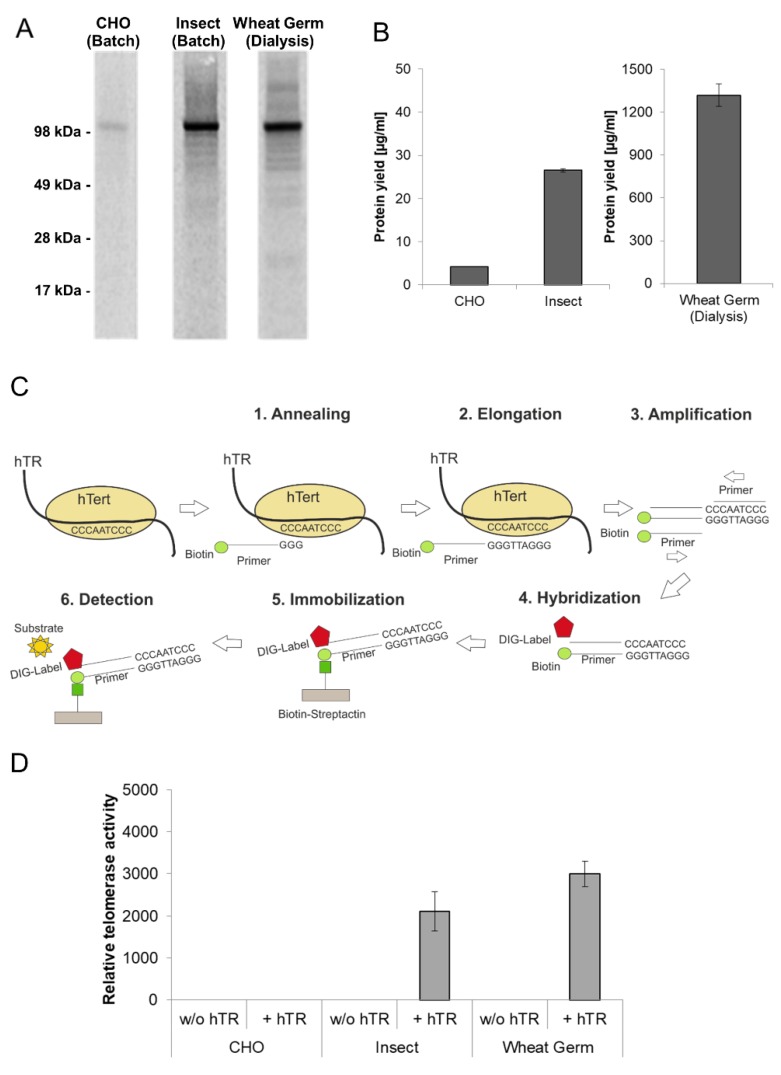
Quantitative and qualitative characterization of cell-free synthesized human telomerase (hTERT). Synthesis of hTERT in eukaryotic cell-free systems in batch (CHO and Sf21) and dialysis (wheat germ) mode. Protein was labeled with ^14^C-leucine for further analysis. (**A**) A 5 µL aliquot of translation reaction mixture was precipitated with acetone and the resulting protein pellets were resolved in sample buffer. Samples were electrophoretically separated on a 10% SDS-PAGE gel followed by autoradiography. (**B**) Determination of hTERT protein yield by scintillation measurement. 5 µL of translation mixture was precipitated using TCA and precipitated protein was soaked on a filter sheet. The filter sheet was dissolved in scintillation liquid and analyzed using an LS6500 multi-purpose scintillation counter (PerkinElmer). (**C**) The general principle of hTERT activity assay (TeloTAGGG Telomerase PCR ELISA PLUS Kit (Roche)). (**D**) Functional characterization of cell-free synthesized hTERT using TeloTAGGG Telomerase PCR ELISAPLUS Kit. Synthesis of hTERT was carried out in the absence and presence of telomerase-specific RNA (hTR). According to the manufacturer’s protocol (TeloTAGGG Telomerase PCR ELISA PLUS, Roche), samples are considered as telomerase-positive if the difference between the sample and the negative control is higher than the twofold value of the negative control. Samples, where the difference is lower than the twofold value of the negative control, were considered as telomerase-negative and set to zero relative telomerase activity.

Telomerase is a ribonucleoprotein which adds hexanucleotides like TTAGGG in vertebrates to the ends of chromosomes and thereby protects the telomeres against aging caused by shortening during cell division. The reverse transcriptase subunit (hTERT) is inactive in most somatic cells but active in nearly all cancer cells showing an unlimited proliferation. For these reasons, it is essential to produce active recombinant telomerase in sufficient amounts for subsequent pharmacological characterization. In vivo expressed hTERT is usually insoluble due to incorrect protein folding. Furthermore, hTERT requires the assembly with a telomerase intrinsic RNA (hTR) to form a completely active ribonucleoprotein. In this chapter, the production of hTERT in cell-free protein synthesis systems was evaluated. Apart from the previously described *Sf*21 and CHO lysate based cell-free system, a third eukaryotic cell-free system based on wheat germ extract was evaluated for the synthesis of hTERT. Protein production using wheat germ lysate differs significantly from previously used systems due to the reaction mode (dialysis mode). The production efficiency and the functional activity of hTERT proteins produced in eukaryotic lysates were compared (Figure 4). In all three systems the presence of a hTERT protein band at an appropriate molecular mass of 127 kDa was detected (Figure 4A). The intensity of the protein band differs in all three systems, which is equivalent to the obtained protein yields (Figure 4B) where large differences were detected (CHO: 3 µg/mL, *Sf*21: 28 µg/mL, wheat germ: 1.5 mg/mL). The highest protein concentration was obtained in the wheat germ dialysis systems with up to 1.5 mg/mL.

As mentioned above, one of the fundamental advantages of cell-free systems is their open nature. Therefore additional components can be easily added to the reaction to improve the quality of the produced protein. In this case, the telomerase intrinsic RNA (hTR) was added in the form of a previously transcribed RNA to CHO and *Sf*21 based batch-systems. Alternatively, hTR encoding plasmid was added to wheat-germ-based dialysis systems. The general principle of the applied TeloTAGGG Telomerase PCR ELISAPLUS Kit (Roche) is illustrated in Figure 4C. Telomerase activity referring to the synthesis of the enzyme in the presence and absence of hTR is given in Figure 4D. 1 µL of each cell-free translation mixture was directly used to perform teloTAGGG assay, hTERT produced in the presence of hTR in *Sf*21 and wheat germ lysates displays functional activity, while the total activity of wheat germ based synthesis is approximately increased by 25% in comparison to *Sf*21 extract based production. With a focus on protein yield, hTERT produced in an *Sf*21 cell-free system showed a significant increased relative activity in comparison to wheat germ hTERT. In contrast to the previously mentioned platforms, hTERT synthesized in CHO cell-free systems showed no activity, but a high background activity could be detected in the negative control (cell-free reaction without synthesized hTERT).

## 4. Discussion

Over the recent years, cell-free protein synthesis was developed to a sophisticated tool for a broad range of applications in research and in the industrial context. Starting in the early 1960s with the first *E.coli* based cell-free system from Nirenberg et al. [11] a huge development of cell-free systems took place until now. Versatile platforms are available based on eukaryotic and prokaryotic cell lysates addressing a broad variety of applications ranging from biomedical areas to biofuel research [10]. A growing demand for proteins is noticeable in the biopharmaceutical industry, which is required on the one hand for the production of novel pharmaceuticals and on the other hand serve as target proteins for the development of drugs. Most of these proteins originate from a human organism or harbor human-like modifications, therefore special needs for the utilized production system are required. Eukaryotic cell-free protein synthesis systems mostly fulfill these requirements by enabling ER based posttranslational modifications and these systems harbor the set of cofactors and chaperones necessary for the correct folding and assembly of human proteins [18]. For a long time, eukaryotic cell-free systems were costly and inefficient, thereby drew limited attention for market applications. However, in recent years, the technology has been improved drastically [20,22,28,38] and achieved a special interest in terms of commercialization. In this article, the production of druggable, mammalian target proteins in eukaryotic cell-free protein synthesis systems is highlighted. Improvement of target protein quality and translation efficiency can be addressed by different strategies starting with the direct improvement of cell lysate by adaptation of cultivation strategies, activation and enrichment of translation-relevant factors and inhibition of protease activities. Various strategies for process optimization are reported in different cell-free systems. For the topic of cell-free reaction conditions, Caschera and Noireaux [39] underlined the importance of preparation of amino acid mixture and relevance of pH and concentration to obtain maximum protein yields in cell-free systems. Apart from this, several articles showed the relevance of precisely adjusted ion concentrations for optimal translation efficiencies [40,41]. Beyond general reaction conditions, optimizations in the area of translation factors are reported for HeLa based cell-free systems. Mikami and colleagues supplemented the mammalian cell-free system with recombinant translation factors eIF4E, eIF2 and eIFB, resulting in an improved protein translation rate [42]. Another approach was the elimination of ribosome inactivation factors in *Bacillus subtilis* and *S. cerevisiae* by genomic disruption which robustly improves the activity of cell-free systems [43]. In this article, the CHO based eukaryotic translation systems were improved by addressing the activation of translation factor eIF2 using the small component inhibitor C38. C38 inhibits the eIF2α specific kinase PERK [44], which phosphorylates the protein and thereby led to inactivation of cap-dependent translation initiation. The results showed that phosphorylation of eIF2α increased during the cell-free reaction, which might be due to ER stress and the presence of unfolded proteins. ER-stress is one of the most relevant factors for activation of ER-based PERK [45] which might be activated by mechanical disruption procedures during lysate preparation. The application of C38 is an efficient method to improve cap-dependent translation initiation in eukaryotic cell-free systems harboring microsomes. The described approach enables a fast and easy improvement of cap-dependent translation initiation independent of the type of eukaryotic cell lysate. By supplementation of C38, a significant improvement of protein yield is obtained. Additional future approaches in the area of eIF2α activation might be performed with a special focus on cost efficiency by directly addressing the cellular signaling pathways. It is conceivable, for instance, to directly engineer the stress response signaling pathway or diminish the expression of PERK in eukaryotic cells, thereby improving the cell-free platform. Similar approaches are already published for *E.coli* based cell-free systems where release factor 1 is depleted to improve incorporation of non-canonical amino acids using amber stop codon technology [46]. In this context, cell engineering strategies are an additional method for the future improvement of eukaryotic cell-free systems.

Optimized cell-free systems can be directly used for the production of druggable target proteins. In this study, WNT proteins and hTERT were exemplarily chosen to be synthesized and functionally characterized in eukaryotic cell-free systems. In conventionally used protein production platforms both proteins can hardly be expressed due to their “difficult-to-express” nature [47,48,49]. The attention for research correlating with WNT signaling is steadily rising mainly because of its fundamental role in the development of numerous diseases [50]. Several diseases are correlated with specific mutations in different components of the WNT signaling pathway [51], while targeted drugs need to be developed to address the mutated proteins. In this study, we have shown the production of different WNT protein ligands in an *Sf*21 based cell-free system. Additionally, we could prove the translocation of the proteins into ER-based microsomes, a requirement for correct posttranslational modifications and correct folding of WNT proteins. The eukaryotic cell-free system showed translocation efficiencies between 20%–40% of total translated protein. Former experiments underline that limitations of translocation are not linked to a limited volume of microsomes [52]. Furthermore, a prolongation of translation reaction by shifting the reaction format to a two-chamber dialysis system led to an increase of translocated membrane protein EGFR up to 60%–70% [22]. During dialysis reaction translation speed is continuously slowed down by gradually decreasing concentrations of energy components which in turn might support a proper ribosomal arrest for assembly of the translocation machinery. An additional factor might be the limited availability of translocation correlated factors like signal recognition particles (SRP) and signal recognition receptors. For cell-based protein production, it is reported that overexpression of SRP led to an increase in antibody secretion [53]. Similar approaches are conceivable for future system improvements. It is noticeable that translocation of WNT proteins into microsomes of *Sf*21 lysate is more efficient using a melittin signal peptide instead of the native human signal peptide of WNT proteins. The compatibility of melittin signal peptide and *Sf*21 cell-free systems was already reported in former studies [54]. The results showed that the eukaryotic cell-free system can not only be used for the production of defined proteins but also for comparison of their signal peptides. This technique paves the way for a future application to evaluate and optimize signal peptide sequences in a fast and high throughput manner prior to protein expression in cell-based systems. In addition to signal sequence optimization, Mel-WNT3a was functionally characterized showing increased biological activity in comparison to commercially available WNT proteins. In eukaryotic cell-free systems, WNT proteins are translocated into ER-derived microsomes and further post-translationally modified by palmitoylation and glycosylation. In eukaryotic cell-free systems, ER based posttranslational modifications are possible due to the presence of microsomes. These microsomes harbor the set of glycosyltransferases present in the ER leading to the formation of core glycosylation, which is identical in various eukaryotic cell-free systems. The presence of glycan moieties on WNT ligands might be beneficial for the correct folding of the protein. Palmitoylation might lead to an anchoring of WNT proteins to microsomal membranes thereby improving their biological activity. Former studies showed an improvement of WNT activity by tethering WNT3a to a liposomal membrane [55]. Water soluble and biologically active forms of WNT3a can also be obtained in the presence of albumin [47]. Eukaryotic cell-free systems with endogenous microsomes proved to be the most efficient platform for the production of biologically active WNT proteins. These results open up new opportunities for the detailed analysis of signaling processes and the characterization of druggable target proteins. Signal transduction recreated in eukaryotic cell-free systems bears an enormous potential for engineering and analysis of cellular pathways. By providing the opportunity to synthesize whole signal transduction pathways in a eukaryotic cell-free system, a fast and comprehensive research tool for multiple stages of signaling pathways has been developed. The open character of the cell-free system enables direct adjustments, modifications and analysis of each step of protein-protein interactions. In combination with high throughput screening approaches, this platform represents a sophisticated module for future drug development. A proof of concept for designing metabolic pathways in cell-free systems is already shown for *E. coli* based cell-free systems addressing the field of industrial biotechnology and biofuel production [56,57,58]. Future developments will focus on eukaryotic cell-free systems to implement this technology into drug development approaches.

In addition to WNT proteins, another therapeutically relevant protein, hTERT, was produced in three different eukaryotic cell-free systems. Ribonucleoprotein hTERT consists of a telomerase protein component (TERT) and a telomerase RNA sequence (TR) harboring only two conserved structural elements, the pseudoknot-template-region and the hairpin structure [27]. To obtain functionally active hTERT, both components need to be available in the protein expression system. The open nature of cell-free protein synthesis enables the direct supplementation of TR, which simplifies the production of the active enzyme. Our results demonstrate that even the cell-free synthesis of complex enzymes is feasible. In this study, two out of three analyzed eukaryotic cell-free systems enabled the production of active telomerase. Noticeable is the high background signal of CHO cell lysate control samples, which might be due to the presence of endogenous TERT in this particular system. Former studies showed that oligodeoxynucleotides containing human telomeric sequences hybridize with telomeres of a huge number of tested vertebrates [59]. Moreover, a cross-hybridization to insect and plant telomeres applying stringent conditions was not observed. Both findings indicate that human telomerase reverse transcriptase can rarely recognize a TR-component that is derived from non-vertebrate organisms. In this context, detectable activity of synthesized telomerase in CHO cell lysates might be possible without applying any additional TR since endogenous TR might be present in these lysates. Diverse production systems for hTERT have been available until now. The synthesis of human telomerase in a eukaryotic cell-free system based on rabbit reticulocyte cells was previously demonstrated by Bachand et al. [60]. The reconstitution of hTERT and a previously transcribed TR resulted in an active telomerase complex. A second alternative, *S. cerevisiae* cells with co-expressed hTERT and hTR, was utilized as a basis to perform functional studies [48]. Both systems provide a powerful tool to study hTERT and hTR interactions in vitro. Nevertheless, for therapeutical approaches which require high yields of active enzyme, both systems have their limitations since relatively low protein yields in the range of several µg/mL were obtained. The use of eukaryotic cell-free systems introduces novel opportunities for the future of hTERT and for the development of potential clinical applications in this area. hTERT is involved in various cancer types [61], therefore further characterization of the hTERT variants and targeted development of drugs may significantly improve cancer treatments.

In this study, the advantages of eukaryotic cell-free systems in the production of druggable protein targets were depicted. Production of target proteins in different eukaryotic cell-free systems was demonstrated showing the diversity of this platform technology. The selection of the type of eukaryotic cell-free system is thereby dependent on the target protein class and the requirement for the desired application. Wheat germ cell-free systems are typically limited in the performance of certain posttranslational modifications [18] and therefore not suitable for complex, modified mammalian proteins, but enable comparable high protein yields. *Sf*21 and CHO extract based cell-free systems allow for the synthesis and modification of complex target proteins and membrane-embedded proteins but are comparatively limited in their productivity. The improvements of eukaryotic cell-free systems in combination with the potential for screening applications and fast and easy protein production opens up new perspectives in future drug development. Initial approaches for the development of miniaturized platforms for high throughput cell-free synthesis are already reported [62,63]. First studies showed screening systems based on *E.coli* and wheat germ cell-free systems [64,65]. Expanding screening technologies for a broad variety of eukaryotic cell-free system will promote future drug development.

## Figures and Tables

**Table 1:** Primer sequences used for WNT DNA template generation including melittin signal peptide (Mel-WNT).

Primer	Primer Sequence 5′ → 3′
Gene-specific forward (F) primers	
X-Mel-WNT3aOpt-F	TAC ATT TCT TAC ATC TAT GCG GAC TCC TAC CCC ATC TGG TGG TC
X-Mel-preWNT5aOpt-F	TAC ATT TCT TAC ATC TAT GCG GAC TTC GCT CAG GTC GTG ATC GAG GC
X-Mel-matWNT5aOpt-F	TAC ATT TCT TAC ATC TAT GCG GAC ATC ATC GGT GCT CAG CCC CTG T
X-Mel-WNT5bOpt-F	TAC ATT TCT TAC ATC TAT GCG GAC CAG CTG CTG ACC GAC GCT AAC TC
X-Mel-WNT6Opt-F	TAC ATT TCT TAC ATC TAT GCG GAC CTG TGG TGG GCT GTG GGT TC
Plasmid specific reverse (R) primer	
pcDNA3-R	CAA AAA ACC CCT CAA GAC CCG TTT AGA GGC CCC AAG GGG AGA AGG CAC AGT CGA GGC TG
Adapter primers	
N-Mel	ATGATATCTCGAGCGGCCGCTAGCTAATACGACTCACTATAGGGAGACCACAACGGTTTCCCTCTAGAAATAATTTTGTTTAACTTTAAGAAGGAGATAAACAATGAAATTCTTAGTCAACGTTGCCCTTGTTTTTATGGTCGTATACATTTCTTACATCTATGCGGAC
C-0	TAATAACTAACTAACCAAGATCTGTACCCCTTGGGGCCTCTAAACGGGTCTTGAGGGGTTTTTTGGATCCGAATTCACCGGTGAT

**Table 2:** Primer sequences used for the generation of eYFP c-terminally fused to Mel-WNT3a (Mel-WNT3a-eYFP).

Primer	Primer Sequence 5′ → 3′
*Gene-specific reverse (R) primer*	
X-Mel-WNT3aOpt-oeXFP-R	CTT GCT CAC CTC TAG ACA GGG CAC CTT TCC AGC G
*eYFP primer*	
X-eXFP-SR	TTG CGG ATG AGA CCA GGC AGA CTT GTA CAG CTC GTC CAT GC
oe-eXFP-F	TGT CTA GAG GTG AGC AAG GGC GA
*Adapter primer*	
C-SII	TGT CTA GAG GTG AGC AAG GGC GA

**Table 3:** Overview of molecular mass of WNT proteins.

WNT Protein	Molecular Mass (kDa) (Human Signal Peptide)	Molecular Mass (kDa) (Melittin Signal Peptide)
WNT 3a	42.9	43.5
WNT 5a	42.3	41.4
WNT 5b	40.3	40.3
WNT 6	39.7	39.3

## Supplementary Materials

The following are available online at https://www.mdpi.com/2409-9279/2/2/30/s1, Figure S1: Cell-free expression of WNT-Signalling Pathway receptor ligands WNT3a, WNT5a, WNT5b, WNT6 based on insect cell lysate and human cell lysat.
