# Supplementary material for: Accelerating the Production of Druggable Targets: Eukaryotic Cell-Free Systems Come into Focus
Source: Methods Protoc. 2019 Apr 16;2(2):30. doi: 10.3390/mps2020030 (PMC6632147; doi:10.3390/mps2020030)
Supplement: Supplementary file 1 [file mps-02-00030-s001.pdf]

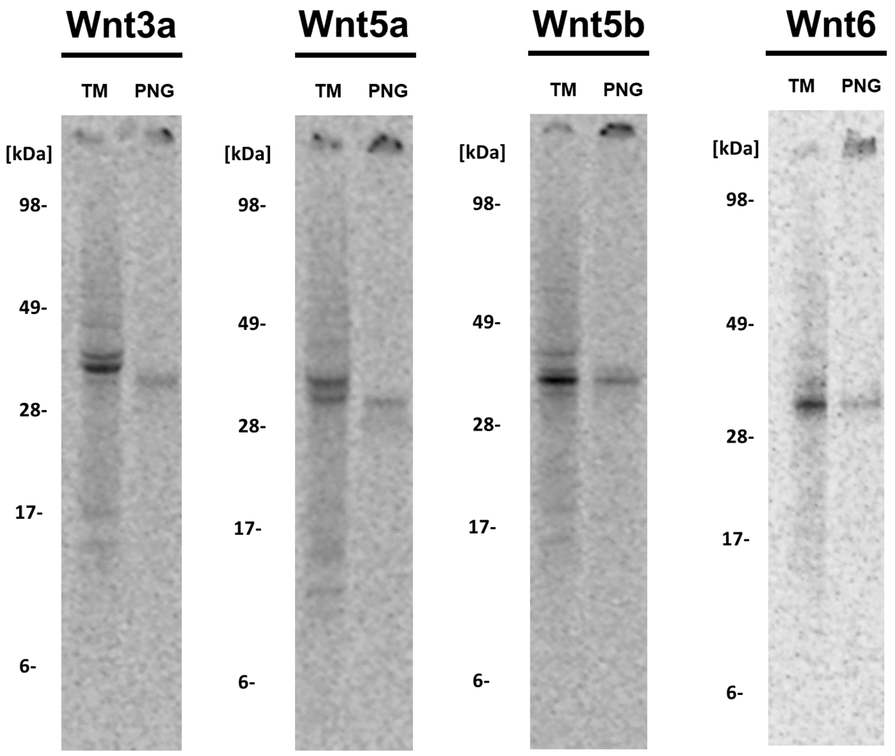

Glycosylation of cell-free synthesized WNT-Signalling Pathway receptor ligands WNT3a, WNT5a, WNT5b, WNT6. WNT synthesis is performed in a linked transcription-translation system in the presence of ^{14}C leucine. Glycosylation analysis of cell-free expressed WNT ligands was performed by PNGase F digestion. An aliquot of 5 µl of radiolabeled protein (Translation mixture (TM) and PNGaseF digested sample (PNG)) was acetone precipitated, applied to SDS-PAGE and analyzed using a phosphoimager (Typhoon TRIO PLUS, Amersham).
